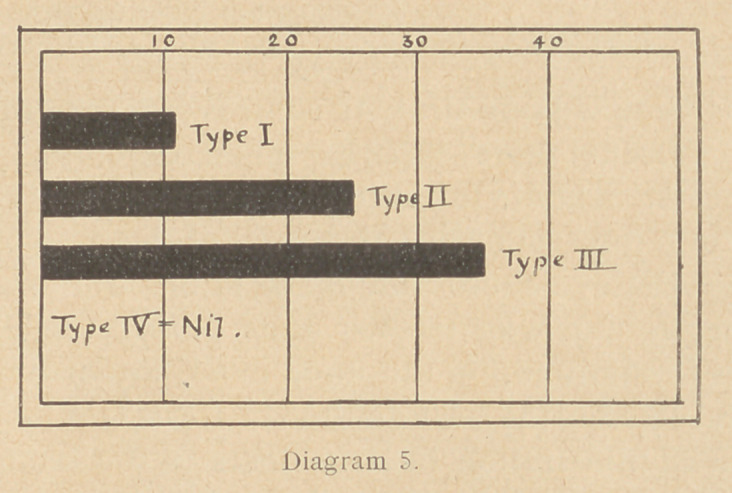# Tetanus

**Published:** 1918-12

**Authors:** David Bruce


### TETANUS

MAJOR GENERAL SIR DAVID BRUCE, C. B., F. R. S.

When your Committee did me the honor to ask me to read a paper before this meeting they suggested the subject of tetanus. Now it is true that I have paid some attention to this disease since the beginning of the war, and in that way the subject is a suitable one. On the other hand, this Society had a discussion on tetanus within the present year, on January 14th and 15th, when Sir William Leishman opened it. That discussion was a very full one, and as the recent papers on tetanus were summarized in the *Medical Bulletin* of January 1918, there remains little to be said on the subject. However, in eight months some slight advance in our knowledge may have been made, and our time may therefore be not altogether misspent if we again review our position.

To begin, allow me to recapitulate some of the facts. It must be strictly understood that these facts only refer to cases of tetanus arising in hospitals in England among the wounded arriving from overseas. They do not deal in any way with cases arising in France.

It may be of some interest to you to see at a glance how many cases of tetanus have occurred in England.

Diagram 1 merely represents the number of cases of tetanus which have been treated in military hospitals in England since the beginning of the war. They are taken from the date of the wound, not from the date of the onset of the disease. For example, in

Diagram 1.

August, 1914, there were 8 men wounded who, sooner or later, developed tetanus. The figures have no relation to the number of wounded or the number of troops engaged. It shows periods of activity and inactivity in the fighting line. For example, the 8 men in August, 1914, were wounded at the battle of Mons, the 54 in September at the battle of the Marne. The cases in October and November were due to wounds received prob-

Diagram 2.

ably at La Bassée and Ypres. The rise in September, 1915, marks the battle of Loos; in July, 1916, the battle of the Somme, and so on.

Diagram 2 is an attempt to give the ratio of cases of tetanus to number of wounded. The question of the incidence of tetanus to wounded cannot be dealt with at all completely at present. This information will probably not be available until several years after the war, but this diagram may be said to give roughly the ratio of the number of cases of tetanus to the number of wounded. From it you will see that the incidence was six times as high in September, 1914, as in November, and nine times as high as in December of the same year. The chief cause in this sudden drop in November was undoubtedly the introduction of the prophylactic injection of anti-tetanic serum which took place about the middle of October, 1914. Why the incidence should have been lower in 1915 and 1917 than in 1916 it is impossible to say, so many different factors came in. Single prophylactic doses were mostly given up to the end of 1916. During 1917 and 1918 multiple prophylactic doses became the rule.

### The Average length of Incubation of Cases of Tetanus Treated in Military Hospitals in England since August, 1914.

Table I gives the average incubation period at different times since the beginning of the war.

From Table 1 it will be seen that the average incubation period has been gaining in length since the beginning of the war. During the first year it was 13.4 days. At the end of 1916 it had risen to 67 days. The average incubation period for all cases since the beginning of the war is 35.9 days.

TABLE I

| ANALYSES | No. of Cases. | Average Incubation in Days. |
|---|---|---|
| 1914–15 | 231 | 13.4 |
| 1915-16 | 195 | 31.2 |
| Aug-Oct. 1916 | 200 | 30.6 |
| Oct.-Dec. 1916 | 100 | 45.0 |
| Dec. 1916 to March 1917 | 100 | 67.0 |
| March-June 1917 | 100 | 44.1 |
| June-Sept. 1917 | 100 | 55.5 |
| Sept.-Dec. 1917 | 100 | 46.9 |
| Dec. 1917-April 1918 | 100 | 46.19 |
| Total | 1226 | 35.94 |

### Variation in the Period of Incubation since the Beginning of the War.

Table II gives the number, in percentages, of cases with short, medium, and long incubation periods, which have occurred since the beginning of the war.

From it, it will be seen that in the first year there were 47 per cent. of cases with a short incubation. This dropped at the end of 1916 to 10 per cent. Since then it has varied betwen 13 per cent. and 26 per cent.

In 1914-15 there were only 6.4 per cent of cases with a long incubation period, but this rose at the end of 1916 to 69 per cent, and still remains high.

This lengthening out of the period of incubation has taken place during the war. Few prophylactic injections of antitoxin were given during the first year, since by far the greater number of cases in that year occurred during the first months, before the administration of prophylactic injections had got under way.

TABLE II

| ANALYSES | INCUBATION PERIODS | | |
|---|---|---|---|
| | Up to 10 days per cent. | 11 to 22 days per cent. | More than 22 days per cent. |
| 1914-15 | 47.0 | 46.4 | 6.4 |
| 1915-16 | 15.5 | 48.8 | 35.6 |
| Aug.-Oct. 1916 | 14.0 | 43.8 | 42.1 |
| Oct.-Dec. 1916 | 12.6 | 26.3 | 61.1 |
| Dec. 1916-March 1917 | 10.0 | 21.0 | 69.0 |
| March-June 1917 | 20.0 | 34.0 | 46.0 |
| June-Sept. 1917 | 15.0 | 24.0 | 61.0 |
| Sept.-Dec. 1917 | 13.0 | 31.0 | 56.0 |
| Dec. 1917-to April 1918 | 26.0 | 29.0 | 45.0 |

### Rate of Mortality

Having discussed the period of incubation, let us now turn to the rate of mortality.

The rates of mortality at different periods of the war are given in Table III. From this Table it will be seen that the fall in the rate of mortality has been progressive. In the first year it was

57.7 per cent., and has since then fallen as low as 15 per cent. in 1917. The total rate of mortality for the 1226 cases is 35.5 per cent.

This rate is, however, too low, since it does not take into consideration the cases which occurred in France, and which were naturally of a severer type than those which occurred in England. Sir William Leishman, in his first analysis of cases in France, based upon 179 cases, gives a case mortality of 78.2 per cent. In a second analysis, in collaberation with Major Smallman, he gave a case mortality of 73.7 per cent. in 160 cases. This would give a total mortality in the 1565 cases in England and France of about 44.3 per cent.

The rate of mortality in pre-serum days was about 85 per cent. Few were expected to recover. In this connection Colonel Harvey has drawn my attention to a statement in Sir Charles Bell's diary, written after the battle of Waterloo. He is describing a case of severe tetanus in a soldier belonging to the German Legion. He says "There are but two cases living of all who had this affection — this man and a French Officer. His death was constantly anticipated by all — and by none more than myself — on account of the general ill-success of our means in these cases". This lowering of the death rate from 85 per cent. to 44.3 per cent. is satisfactory, and is doubtless due in great part to the introduction of the prophylactic inoculation of antitoxin. It must also be borne in mind that, so far as prophylactic injections were concerned, we were still practically in the pre-serum days during the first months of the war.

TABLE III

| ANALYSES | N° of Cases. | Recovered. | Died. | Mortality per cent. |
|---|---|---|---|---|
| 1914–15 . . . . . . . . . . | 231 | 98 | 133 | 57.7 |
| 1915–16 . . . . . . . . . . | 195 | 99 | 96 | 49.2 |
| Aug.-Oct. 1916 . . . . . . | 200 | 127 | 73 | 36.5 |
| Oct.-Dec. 1916 . . . . . . | 100 | 69 | 31 | 31.0 |
| Dec.-March 1917. . . . . . | 100 | 81 | 19 | 19.0 |
| March-June 1917. . . . . . | 100 | 71 | 29 | 29.0 |
| June-Sept. 1917 . . . . . | 100 | 85 | 15 | 15.0 |
| Sept.-Dec. 1917 . . . . . . | 100 | 84 | 16 | 16.0 |
| Dec.-April 1917-18. . . . . | 100 | 76 | 24 | 24.0 |
| Total. . . . . . | 1226 | 790 | 436 | 35.5 |

### Types of Tetanus.

At the beginning of the war, only the proportion of cases of local tetanus to general tetanus was recorded. Since June, 1917, a more detailed classification has been attempted. Since that date there have been 223 cases of general tetanus, and 77 cases of local tetanus.

Table IV gives the various Types. From it, it will be seen that there is a mortality of 58.7 per cent. in cases with complete closure of the jaws developing within 24 hours after the onset of symptoms. This is the highest mortality, and may be compared with cases of general tetanus without trismus in which the rate of mortality is only 15.7 per cent., and local tetanus, in which the rate of mortality is nil.

TABLE IV

| | Cases. | Recovered. | Died. | Mortality per cent. |
|---|---|---|---|---|
| (A) Trismus the earliest symptom. | | | | |
| (1) With complete closure of jaws developing within 24 hours after onset of symptoms . . . . . . . . | 17 | 7 | 10 | 58.7 |
| (2) With complete closure of jaws, developing after 24 hours . . . . . . . . . | 19 | 14 | 5 | 26.2 |
| (3) With incomplete closure of jaws . . . . . . . . . . . . | 111 | 85 | 26 | 23.4 |
| (B) Trismus occurring after other symptoms of tetanus have shown themselves . . . . . | 57 | 46 | 11 | 19.2 |
| (C) General tetanus without trismus. . . . . . . . . . . . | 19 | 16 | 3 | 15.7 |
| (D) Local tetanus. . . . . . . . . | 77 | 77 | 0 | 0 |

Now having brought these figures to your notice, the question arises as to what are the most important points to be considered in the prevention and treatment of tetanus.

### (a) *Preventive Measures.*

These points are :

(1)—The best surgical procedure to prevent tetanus.

(2) The importance of the early prophylactic injection of anti-tetanic serum in trench foot.

(3) The best dosage in prophylactic inoculations.

(4) The benefit, if any, accruing from multiple prophylactic injections.

(5) The addition of anti-gas-gangrene and anti-vibrion septique serum to anti-tetanic serum.

(6) Types of tetanus bacilli.

b) *Therapeutic Measures.*

(1) Route of injection.

(2) Dosage.

TREATMENT OF TETANUS BY ANTI-TETANIC SERUM.

A. Prophylactic or Preventive Measures.

(1) *What is the best surgical procedure in cases of gun-shot wounds in order to prevent the supervention of tetanus?* In the 2nd analysis of cases of tetanus in 1915, it was said that if thorough surgical treatment is carried out on wounds from the beginning, so as not to allow the presence of necrotic tissues or foreign bodies, the number of cases of tetanus should sensibly diminish, if not altogether disappear.

The surgeon's knife, after all is said and done, is the best means of preventing the occurrence of tetanus. It stands in the first rank as prophylactic. Dead putrifying tissue is the home, the favorite environment of the anaerobe. Place washed tetanus spores among healthy living tissues and there is nothing doing. Add a trace of gas-gangrene toxin, or a chemical irritant, such as sapronin, or a physical irritant, such as earth or any foreign body, and the tetanus bacilli have their tails up at once. At the beginning of the war the treatment of wounds was not thorough enough at the primary operation. It was thought sufficient to wash out the wound and apply an antiseptic. Lately, however, the thorough excision of wounds has come more and more into vogue. That this has had a good effect is borne out by Capt. Tulloch and Miss Cayley's work at the Lister Institute. They have shown that in wounds which have been submitted to a wide resection at the primary operation, the number of anaerobes is very much less — they are in fact often difficult to find — than in wounds treated by the older methods.

Another argument in favor of this thorough surgical treatment of wounds is brought forward by Sir Kenneth Goadby and others.

They make it clear that many kinds of bacteria may lie latent in wounds for months or even years, and that complete healing of the wounds may take place in spite of their presence. These collections of bacteria lying in the depths of scar tissue, or sequestrum of bone, may give rise to no inconvenience, but, on the other hand, some slight accident such as a fall, or a plastic operation undertaken months after the wound has healed, or even the ordinary operation of massage, may release them and start an attack of tetanus months or even years after the wound was received. Naturally it is the resistent spore bearing anaerobic bacteria, such as tetanus and gas-gangrene, which persist longest in the wounds; in fact, as Goadby has shown, end-sporers similar to tetanus bacilli have been found in bone sequestrum as late as three years after the infliction of the wound.

You will agree with me, then, that the first and most important measure in the prevention of tetanus is the thorough surgical treatment of the wound at the primary operation.

*Antiseptic.* In regard to antiseptics, none of them have given very encouraging results. No antiseptic is known which has the power of totally destroying micro-organisms in wound exudates, without at the same time exciting a markedly deleterous influence on the tissues. Of course, any antiseptic used effectively will reduce the infection of wounds. The good results given by any particular antiseptic depend rather on the surgeon than the solution.

(2) *The importance of the early prophylactic injection of antitetanic serum in trench foot.* On January 13th, 1917, a paper on "The Importance of the Early Prophylactic Injection of Antitetanic Serum in Trench Foot" was published in the *British Medical Journal.* As this gave some interesting results it may be useful to recall your attention to it. The paper stated that:

"Cases of tetanus following on "trench foot" have lately been numerous. On account of the hurt not being of the nature of an ordinary gun-shot or shell wound they have not, until quite lately, received the usual prophylactic dose of antitetanic serum at the front.

"During the last few weeks fifteen cases of tetanus caused by "trench foot" have been reported, but full reports, giving the result, have only been received in eight. The following table gives the outstanding features of these eight cases. A prophylactic inoculation was not given in any of them.

TABLE V

| No | Incubation. | Duration of Disease. | Died. | Recovered. |
|---|---|---|---|---|
| 1 | 12 days | 2 days | D | |
| 2 | " " | | | R |
| 3 | 24 " | 2 " | D | |
| 4 | ? | 2 " | D | |
| 5 | 15 days | 1 " | D | |
| 6 | ? | 1 " | D | |
| 7 | 16 days | | | R |
| 8 | 14 " | 5 " | D | |

"From this table it will be seen that of the eight cases only two recovered, and the average duration of the disease was only 2.5 days. This is a return to the picture of tetanus, familiar before prophylactic injections were introduced. The disease wastes no time in local manifestations, but bursts out as generalized tetanus and runs its acute and fatal course in twenty-four to forty-eight hours.

"It is to be hoped that no medical officer in charge of a case of "trench foot" will hesitate for a moment in giving a prophylactic injection of antitetanic serum, and repeating the same at intervals of seven days until the wounds are clean. If something is not done speedily, these cases of tetanus following on "trench foot" will run up in a most disastrous way the rate of mortality in tetanus, which every one, by the use of prophylactic and early therapeutic antitetanic serum, is trying to lower."

(3) *What is the best dose of antitoxin to give as a primary prophylactic inoculation?* The Tetanus Committee in the third edition of their Memorandum recommended a primary injection of 500 U. S. A. units. Lately, Colonel Cummins, the Advisor in Pathology to the B. E. F. in France, has come to the conclusion that this dose is too small, and has recommended that it be increased to 1500 units. Instructions for this change in dosage have already been issued by the War Office.

Colonel Cummins states that several severe and fatal cases have lately occurred in which the smaller dose had been given. It is to be feared that a certain number of these severe and fatal cases will always occur in spite of any dose of antitoxin. In infection experiments on animals, if the destruction of tissue exceeds a certain limit, no amount of antitoxin will save the animal, and so it probably is with man.

Colonel Cummins gives the following reasons for the change.

1. First he gives a table representing the Rate of Mortality in cases of tetanus, which have received different primary prophylactic doses of serum.

TABLE VI

| UNITS | N° of Cases. | Rate of Mortality. |
|---|---|---|
| 500. . . . . . . . . . . . . . . . . . . . . . | 135 | 74.1 |
| 750-1000 . . . . . . . . . . . . . . . . . . | 40 | 55.0 |
| 1001-1500. . . . . . . . . . . . . . . . . . | 14 | 50.0 |

2. He notes that the number of cases receiving 750-1000 and 1001-1500 units is very small, although an instruction was sent out on the 4th of July 1917, recommending that a dose of 1000-1500 units should be given " in all deep wounds, in those which are contaminated with dirt, and in those in which there is fracture of bone ". From this he draws the deduction that the small number of cases in which tetanus has supervened in persons to whom these larger doses had been given suggests that the prophylactic effect must be considerably greater than where the smaller doses are used.

3. He goes on to say that while the figures tabulated in Paragraph 1 are suggestive, it must be noted that the total numbers are very small, and perhaps insufficient to justify final conclusions. In the absence of larger statistical evidence, the theoretical reasons for advocating a larger initial prophylactic dose appear to him to be very strong.

(*a*) It appears justifiable to assume that the larger the initial subcutaneous injection, the longer the period of absorption, and the longer the time during which an effective amount of antitoxin will still be present in the body fluids.

(*b*) This prolongation of antitoxic effect should, theoretically, assist in tiding the wounded man over the dangerous period of shock and sepsis, and in giving the human organism more time in which to augment its immune substances against the tetanus bacilli implanted in its tissues.

(4) He goes on to say that, while it is unquestionably true that the administration of 500 units as a prophylactic inoculation has been of great service in diminishing the incidence of tetanus amongst the wounded, it is still reasonable to suppose that there is a quantitative relation between the degree of infection in any given wound and the amount of available antitoxin that is necessary to prevent or modify an attack of tetanus.

The more heavily infected wounds should, theoretically, require larger doses than would suffice for wounds less grossly contaminated.

(5) In this connection, he thinks it worth while making the following quotation from an article on tetanus by Dr. A. MacConkey, of the Lister Institute. " Where there is plenty of serum at the disposal of the surgeon, there is no need to take thought about the size of the dose; and the ample dose of 1000 to 1500 units may be given with the knowledge that it is better to give too much than too little. "

Now it is difficult, if not impossible, to bring effective arguments to bear in favor of a 500 unit dose against a 1500 one. In all probability it is true that 1500 is rather better than 500 as an initial dose, and especially if only one is given. But the arguments brought forward by Colonel Cummins are unconvincing. The recommendation of the Tetanus Committee was four injections of 500 at intervals of seven days. Colonel Cummins' figures give no evidence at all that this is insufficient, nor do they afford any grounds for altering the Committee's recommendation.

In his first paragraph he gives a table which is suggestive, but the numbers are too small to give any satisfaction. I have made out a similar table for the English cases. There are 271 cases recorded as having only received a single prophylactic dose of 500 units. These give a rate of mortality of 30.6 per cent. There are only 18 cases which received from 750 to 1000 units. They give a mortality of 27.7 per cent. There were only two cases which received a single prophylactic dose of 1500. One recovered and one died. These figures are, of course, too small, but they do not help Col. Cummins' argument.

Paragraph 2 is also a wipe-out as there are no figures available.

Paragraph 3 (*a*). His assumption does not appear to me justifiable. Levin has shown that it makes no difference whether 10, 20, or 40 cc. of heterologous serum be injected into a rabbit; practically all has disappeared at the end of six days. The evidence in tetanus seems also to be that it is just the same whether you inject 500 or 1500 units; the antitoxins have vanished by about the tenth day. There is some evidence that if very large doses are given, some lengthening of the passive immunity can be brought about. MacConkey writes " calculating from the guinea-pig figures, a man weighing 70 kilos. would require some 35,000 units of antitoxin to protect him for one month against a fatal dose of tetanus toxin" .

Paragraph 3 (*b*). There is no evidence of prolongation of antitoxic effect with the 1500 units dose, and if the man receives his second prophylactic dose at the end of seven days there is no necessity for any prolongation. In regard to the second argument in the same paragraph, it is well known that as long as the blood contains antitoxin the human organism will do nothing to augment its immune substances. In order to get the organism to help itself towards active immunity, it is essential to give the least possible amount of antitoxin just enough to prevent the toxin causing death. As the toxins tend to rise to the level of the antitoxins, the organism is driven to supplement the supply by home-made antibodies.

In regard to paragraph 4, my contention is that as toxin is, as a rule, formed slowly and in small quantities, the 500 units weekly are enough and more than enough to neutralize what is formed.

In paragraph 5, Col. Cummins quotes MacConkey as an argument in favor of a 1500 unit dose. But MacConkey also says " If the dose of 500 units is repeated at the end of a week, and of a fortnight, after the receipt of the wound, we should not use any more serum, and we should probably get a more prolonged immunity than by giving the 1500 units which some surgeons seem to prefer ". It is therefore evident that quotations won't help us much.

The Tetanus Committee after taking all these points into consideration, also the questions of transport, cost of serum, etc., involved, were of opinion that 500 units was the best all-round dose for the four prophylactic injections.

I, for one, remain of the same opinion, but the Tetanus Committee are content to compromise in this matter. Moreover nothing would be gained by holding to their previous conviction, as the authorities have already made the change. The primary injection recommended to be given at the front, has hitherto been 500 units, a dose which in their opinion has proved on the whole adequate. It has recently been raised to 1500 units in the hope that an even higher degree of immunity may be conferred. The Committee sincerely hope that the experiment will be successful. It is understood that the second, third, and fourth injections remain at 500 units.

4. *Does any benefit accrue from multiple inoculations?* The Tetanus Committee stated that the prophylactic value of injections of anti-tetanic serum is beyond all doubt, but that there is strong experimental evidence that in about ten days the immunity conferred by an injection is to a great extent lost. The Committee therefore recommend that a second, third, and fourth subcutaneous injection should be given to all wounded men, if possible at intervals of seven days. This recommendation was made in their Memorandum of October, 1916, and also in that of June, 1917, so that multiple injections have been in vogue more or less since the beginning of 1917. At present the number of our wounded who receive four inoculations, are very various depending on the energy and belief of the local authority. It may be said to vary between 40 per cent. and 90 per cent.

What one would principally expect from multiple inoculations would be a lowering of the rate of the incidence of tetanus among the wounded. One would not look so much for a lowering of the rate of mortality in cases which had actually occurred. Is there any evidence, that a lowering of incidence has taken place? The only evidence which can be brought forward is shown in Diagram 2. Here it is evident that the incidence in 1917 has fallen as compared with 1916. Is this due to multiple injections or to more thorough primary resections of the wounds by the surgeons, or to what? With our present knowledge I am afraid it is impossible to say.

The result as regards the Rate of Mortality up to the present is given in the following Table.

TABLE VII

| Nº of injections. | Nº of Cases. | Recovered. | Died. | Mortality per cent. |
|---|---|---|---|---|
| 1 . . . . . . . . . . . . . . | 201 | 157 | 44 | 21.9 |
| 2 . . . . . . . . . . . . . . | 166 | 133 | 33 | 19.8 |
| 3 . . . . . . . . . . . . . . | 65 | 53 | 12 | 18.5 |
| 4 . . . . . . . . . . . . . . | 43 | 39 | 4 | 9.3 |
| 5 . . . . . . . . . . . . . . | 15 | 14 | 1 | 6.6 |
| 6 . . . . . . . . . . . . . . | 2 | 2 | " | " |

Here we see a gradual fall from 21.9 to 6.6 per cent. But again is this due to the multiple inoculations, or only to the fact that a patient who does not show symptoms of tetanus for four weeks probably belongs to the class with a long incubation period. A long incubation, as we have seen, gives a low rate of mortality. Unfortunately the data collected up to the present are insufficient to decide the matter.

You must judge for yourselves if it is worth while to continue multiple prophylactic inoculations. The Tetanus Committee still recommend them. They cannot, as far as can be seen, do any harm, and until our knowledge of the immunizing process in tetanus is more advanced, the Committee think they ought to be continued.

At this point I should like to say that just as there has been a unification of the Military Command at the Western Front, so there ought to be a unification in regard to such things as the treatment of tetanus. For the sake of avoiding overlapping, to ensure some continuity of policy and to make the various data comparable, the prophylactic and therapeutic treatment should be run if possible on similar lines.

Small tetanus committees of the American, British, and French Armies could be associated with the Tetanus Committee in London. The Tetanus Committee would forward all information at their disposal to the other Committees and would receive from them reciprocal treatment. Then there might be some chance of escaping overlapping, and obtaining continuity of policy.

What is really wanted is an International Pathological Committee. A move on in the right direction was brought about when the American Red Cross Society set up a Pathological Research Committee and their Medical Bulletin.

(5) *Is any benefit likely to accrue from the addition of gas-gangrene and vibrion-septique sera to the anti-tetanic serum?* It is only since the outbreak of the present war that the importance of gas gangrene, both as a clinical entity and as a factor in the causation of tetanus, has been appreciated. To Dr. Weinberg of the Pasteur Institute, and to Major Carrol Bull, U. S. M. C., we are indebted for the greatest advance that has so far been made in the prevention and treatment of this disease.

The thanks of bacteriologists are due to Dr. Weinberg for evolving order out of chaos in his study of the anaerobic infections. By drawing attention to the multiplicity of these, and, what is more important, by suggesting how they may influence one another, he has given us a new point of view from which to regard these infections.

In England our interest in the subject was especially stimulated by a visit from Major Bull in the spring of the present year. Major Bull gave a demonstration at the Royal Army Medical College, London, on his method for the production of the toxin of gas gangrene (B. Welchii). Dr. O'Brien, a member of the Tetanus Committee, at once set to work to immunize horses and prepare a double serum. By the beginning of March he had 100 horses producing this serum. Capt. Tulloch also did some work on behalf of the Committee and showed that there is good ground for believing that the auxiliary part played by B. Welchii in the causation of tetanus is clearly defined, but that the power of this anaerobe for doing harm in this direction can be nullified by the use of its own antitoxin. He further shows that the toxin of Vibrion septique is also an ally of the tetanus bacillus. He is of opinion that antibodies to the toxins of B. Welchii and Vibrion septique should be included in all sera employed for the prophylaxis of tetanus. He however utters the warning that although such a polyvalent serum promises to reduce still further the incidence of tetanus, there are still other infective agents such as Bacillus oedematiens which may also play a part in stimulating the growth of the tetanus bacillus in wounds.

On March 5th, 1918, the Tetanus Committee recommended to the Director-General, Army Medical Service, to put this double serum into use as soon as possible, so that its value might be put to a practical test. On March 28th 1000 phials of the new serum were sent to France, and a further 9000 on March 30th. By the middle of July 60,000 doses had gone over.

In regard to the preparation of this double serum credit is also due to Miss Robertson of the Lister Institute, to Dr. Mc Intosh of the London Hospital, and Captain Henry, R. A. M. C., the two latter working on behalf of the Medical Research Committee.

On July 27th, 1918, at a War Office conference in London, it was decided to add the antitoxin of Vibrion septique to the double serum. This triple serum is to contain in each dose 1500 units tetanus, 250 units gas gangrene, and 2500 units Vibrion septique antitoxins. No official report has been received by the Tetanus Committee, but it is rumored that the double antitoxin has not been a marked success up to the present in the prevention of gas gangrene. As to the usefulness of the triple serum, no information is as yet available.

It is then too early to give opinion as to what benefit will accrue from the addition of these two sera to the tetanus antitoxin.

(6) *Types of tetanus bacilli.* Capt. Tulloch, on behalf of the Tetanus Committee, has been hard at work since September, 1916, studying the tetanus bacillus. He has separated it into four types or varieties by means of agglutination tests. That is to say, he has prepared a serum for each of the four types, and uses these sera to identify and separate them.

He has examined 100 cases of tetanus, in which the organism was obtained in a sufficient condition of purity, to permit of its being classified by serological tests.

Let us now turn our attention for a moment to the result of the examination of wounds in men not suffering from tetanus. Up to the present, twenty-three strains of the tetanus bacillus have been obtained from such cases. Seventeen belonged to type I, three to type II, two to type III, and one to type IV.

Diagram 3.

Now for purposes of comparison diagram 4 has been made to show percentage incidence of each type from cases of tetanus, and from cases of wounded men not showing symptoms of tetanus.

From this diagram it is seen that only 41 per cent. of the cases

Diagram 4.

Types of B. tetani obtained from wounds of men suffering from tetanus, compared with bacilli of the same types obtained from wounds of men not suffering from tetanus, expressed as a percentage of the total number of each type isolated from each source.

of tetanus among our wounded is attributed to type I, whereas 74 per cent. of the tetanus bacilli obtained from non-tetanus cases belong to this type.

This is interesting when we consider that up to the present the antitoxin prepared in England is prepared from type I bacilli. If the sera were specific, then we would expect to find that type I serum would neutralize the toxin elaborated by type I bacilli to a greater extent than it would neutralize the toxins of types II, III and IV.

But Capt. Tulloch has shown that anti-tetanic serum is not specific, that the toxin produced by the four types is the same, and any one of the four antitoxins will neutralize all or any of the four toxins.

It would therefore appear that it is only necessary to inject horses with toxin of any type in order to produce effective antitoxin for all, but in order that there will be no mistake, it is usual in England at the present time to inject the horses with toxins from all four types.

But although the antitoxic function of the serum may be the same for all, may there not be other functions also present, such, for example, as anti-bacterial, the function of hindering the growth of, or destroying the bacilli themselves.

It has been shown by Capt. Tulloch that in the case of phagocytosis, for instance, the immunization of an animal with the whole living culture of one type of the bacillus leads to the production of phagocyte stimulating antibodies which are specific to each type. He thinks that sera possessing such extra antibodies may be more efficient in preventing infection than sera which lack them. The ordinary anti-tetanic sera prepared by the inoculation of toxin alone does not possess this phagocyte stimulating property. Much remains to be done along these lines, and I have only brought them forward in order, if possible, to stimulate enquiry.

It has then been shown that there are four types of tetanus, and that these types occur in various proportions in the wounds of men suffering from tetanus, and also in the wounds of men showing no symptoms of tetanus. It has also been suggested that neutralization of the spasm-producing toxin of B. tetani may not be the only factor, in the passive immunity to the disease, set up by prophylactic inoculations.

Let us now consider the virulence of the four types. Is the rate of mortality the same in the four types, or is the disease arising from another type?

The following diagram gives the Death Rate expressed as a percentage of the number of cases in which each type of the bacillus was isolated from men suffering from tetanus. All the cases had received prophylactic inoculations of anti-tetanic serum.

From the diagram it is seen that the death-rate from all types of the tetanus bacillus among inoculated men in whose wounds

Diagram 5.

the bacilli were found is twenty-two per cent.; in type I eleven per cent., type II twenty-five per cent., and type III thirty-five per cent. In type IV all the cases recovered.

### B. Therapeutic Measures.

Whereas there can be no shadow of doubt as to the benefit of prophylactic injection of antitoxin, we are on much less firm ground when we come to consider antitoxin as a curative agent. In fact there does not seem to be any statistical evidence that serum given therapeutically has marked effect on the rate of mortality. Captain Golla, a member of the Tetanus Committee, comparing modern practice with those of pre-serum days, shows that the mortality in cases of tetanus occurring in this war, which did not receive a prophylactic injection, but were treated therapeutically with serum, approaches very closely to the rate of mortality in pre-serum days. It does not appear that the therapeutic, as distinguished from the prophylactic power of serum, is of any efficacy: —

It seems to be admitted that tetanus toxin which has been taken up and fixed by the nerves or nerve cells is inaccessible to antitoxin. If a lethal dose has been taken up by the nerves and is travelling towards the nervous centres, before the serum treatment is begun, then no amount of antitoxin will save the patient.

But in spite of all these considerations, we must give our men the benefit of the doubt and use serum therapeutically. As Ransom says, it may neutralize some of the free toxin in the blood and lymph, and prevent its ultimately entering the nervous system and causing death, when the toxin already admitted through the motor nerves is not sufficient to do so.

It is then up to us to decide on the best route and the best dosage.

*What is the best route for the injection of anti-tetanic serum?*

There has been a good deal of controversy regarding the best route. At present there is also much difference of opinion. The Manchester School, for example, recommends *one* large dose of 30,000 units intravenously, given under deep chloroform anesthesia. The Netley School pin their faith on the subcutaneous route, giving as much as 100,000 units during the first 24 hours. The Tetanus Committee, on the other hand, are of opinion that in acute general tetanus the best method of treatment lies in the earliest possible administration of large doses of antitetanic serum by the intrathecal route. The Committee agrees that the intravenous route is an excellent and rapid one, but think that the extra danger from anaphylactic shock puts it out of court. Professor Dean in his paper gives fourteen cases with only one death. On the other hand, I find that ten of the last twelve cases reported to me, which had large intravenous injections, died, and six of the deaths occurred soon after the intravenous injection. Anaphylactic shock is given as the cause of death in three cases. The result of animal experiment, as shown by Sherrington, is so much in favor of the intrathecal route that the Committee see no reason for changing their opinion. They therefore still recommend the intrathecal route in all cases of generalized tetanus. In cases of local tetanus the intramuscular is considered sufficient.

*What is the best dosage?*

The Tetanus Committee still recommend that in the treatment of acute general tetanus large doses should be employed. As an example of a large dose, the Committee cited 24,000 units on the first and second day. Lately a circular has been promulgated by the British Authorities in France, advising much larger doses. The circular states that the treatment of generalized tetanus by means of antitoxin has not so far given encouraging results, and that in a recent series of 60 cases the case mortality was 71.2 per cent. The dosage of 24,000 units on the first day, recommended by the Tetanus Committee, is looked upon as too small, and a dosage three times as large is advocated. Three cases are given in which recovery took place after very large doses. The first received 64,000 units during the first 24 hours, given in three intrathecal, 1 intravenous, and 4 subcutaneous injections. Eight different injections in the first 24 hours, and all of 8,000 units! This may be necessary, but it is difficult to picture such a quantity of *toxin* circulating in the blood and lymph as to require such a huge quantity of antitoxin for its neutralization. The first two cases had severe serum rashes, and the second is reported to have had a moderate anaphylactic shock. I cannot believe that such numerous and large doses of serum are necessary; on the contrary I believe in many cases they will be harmful. If symptoms of tetanus appear, it is necessary to flood the blood and lymph with antitoxin. Animal experiments show that the intrathecal route is the most efficacious. One would therefore think that a large dose of serum given intrathecally on the first and second days, supplemented and continued by intramuscular and subcutaneous injections, would be sufficient to keep the blood amply supplied with antitoxin. But our knowledge of what really happens in the body, concerning the behavior and action of toxins and antitoxins, is so crude and rudimentary that it may well be that larger doses of antitoxin are required. The result of these enormous doses will therefore be awaited with interest.

But the opinion of the Tetanus Committee is still that a reasonable and suitable dosage is about 16,000 units on the 1st and 2nd day, given intrathecally, and about 8,000 units, intramuscularly.

### PAPERS BY MEMBERS OF THE TETANUS COMMITTEE

Andrewes, Major. F. W.

"On the Intrathecal Route for the Administration of Tetanus Antitoxin", *Lancet*, 5.5.17.

Bruce, Surgeon-General Sir David.

"Note on the Incidence of Tetanus among Wounded Soldiers", *British Medical Journal*, 27.1.17.

"Importance of Early Prophylactic Injections in Trench Foot", *British Medical Journal*, 13.1.17.

"Intramuscular V Intrathecal Injections", *Lancet*, 5.5.17.

"1st Analysis of Cases of Tetanus Treated in Home Military Hospitals from August 1914 to August 1915", *Lancet*, 23.10.15.

"2nd Analysis, From August 1st, 1915, to July 31st, 1916", *Lancet*, 2.12.16.

"3rd Analysis, August-October 1916", *Lancet*, 30.6.17.

"4th Analysis, October-December 1916", *Lancet*, 15.9.17.

"5th Analysis, December 1916-March 1917", *Lancet*, 22.12.17.

"6th Analysis", *British Medical Journal*, 16.3.18. (Summary only).

"Tetanus in Home Military Hospitals — Analysis of 1000 cases", *Transactions of Soc. Trop. Medicine and Hygiene*, November 1917.

"7th, 8th, and 9th Analysis in American Research Committee's library."

Goadby, Sir. K.

"The Bacterial Flora of War Wounds", *British Medical Journal*, 25.5.18.

Golla, Capt. F.

"A Comparison of Subcutaneous with Intravenous and Intrathecal Administration of Tetanus Antitoxin in Experimental Tetanus", *Lancet*, 5.5.17.

"Analysis of Recent Tetanus Statistics", *Lancet*, 29.12.17.

MacConkey, Dr. A. T.
"The Keeping Qualities of Therapeutic Serums", *Lancet*, 6.1.17.
"The Prophylaxis of Tetanus", *British Medical Journal*, 11.12.15.

Sherrington, Prof.
"Observations with Anti-tetanic Serum in the Monkey", *Lancet*, 29.12.17.

Smallman, Major. A. B. (with Col. Sir. Wm. Leishman).
"An analysis of Recent cases of Tetanus in the B. E. F.", *Lancet*, 27.7.17.

Tulloch, Capt. W. J.
"Concomitant Anaerobic Infections in Tetanus", *British Medical Journal*, 1.6.18.
"The Isolation and Serological Differentiation of B. Tetani", *Proc. Royal Society*, Series B., April 1918.

Ransom, Dr. F.
"A Modern View of Tetanus", *Lancet*, 22.12.17.

OTHER PAPERS

Barnes, Capt. Stanley.
"Tetanus Relapse after a Trivial Operation", *British Medical Journal*, 17.3.17.

Dean, Major. H. R.
"A Report on 25 cases of Tetanus", *Lancet*, 5.5.17.

Gibson, Major. A. G.
"A Note on the Reflexes in Tetanus", *Lancet*, 15.9.17.

Worster-Drought, Capt. C. G.
"Observations on a Severe Case of Tetanus Treated with Repeated Intrathecal Injections of Antitoxin", *Royal Army Medical Journal*, January 1918.
"A case Illustrating an Extreme Modification of Local Tetanus", *Lancet*, 1.6.18.

MEMORANDA PUBLISHED BY THE WAR OFFICE.

Memorandum on Tetanus (Third Edition).
Memoradum on the Use of Curative Sera. Anaphylaxis.
Notes on the Nursing of Cases of Tetanus.

## Figures and Tables

**Diagram 1. f1:**
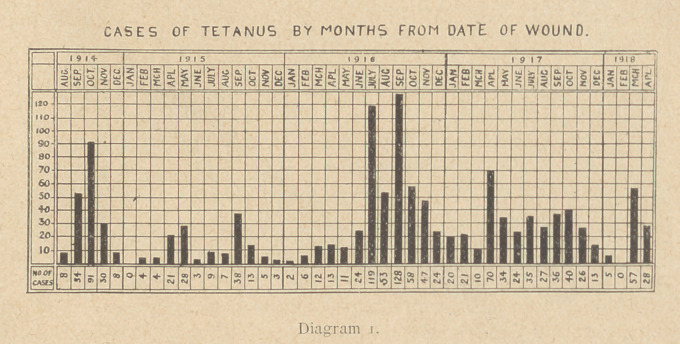


**Diagram 2. f2:**
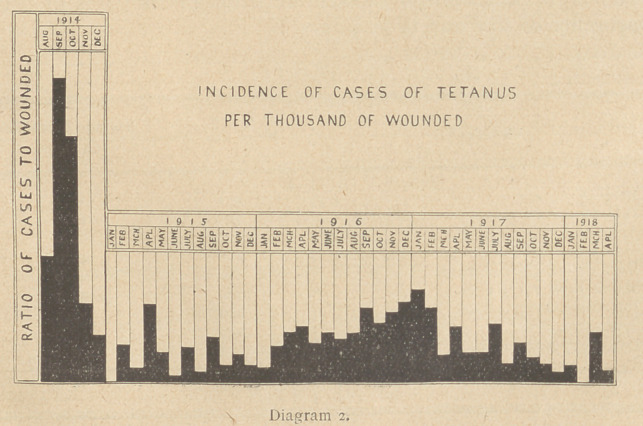


**Diagram 3. f3:**
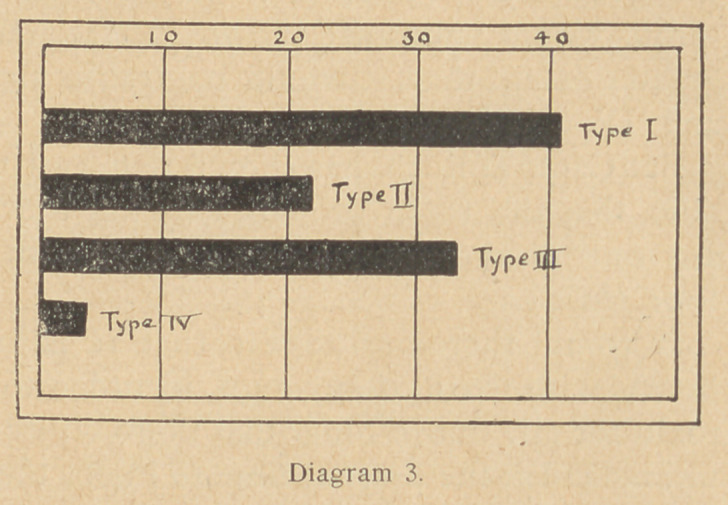


**Diagram 4. f4:**
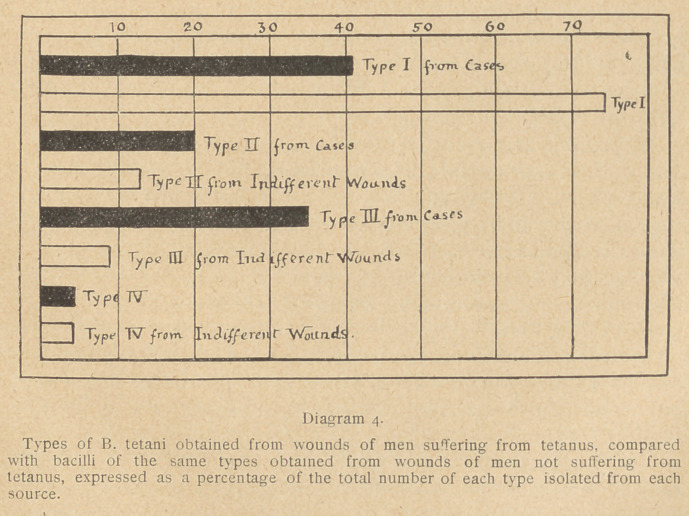


**Diagram 5. f5:**